# Experimental and computational chemical studies on the corrosion inhibition of new pyrimidinone derivatives for copper in nitric acid

**DOI:** 10.1038/s41598-022-20306-4

**Published:** 2022-09-27

**Authors:** A. S. Fouda, M. A. Ismail, M. A. Khaled, A. A. El-Hossiany

**Affiliations:** 1 Department of Chemistry, Faculty of Science, Mansoura University, Mansoura, 35516 Egypt; 2 Basic Science Department, Faculty of Engineering, Horus University, New Damietta, Egypt; 3 Delta for Fertilizers and Chemical Industries, Talkha, Egypt

**Keywords:** Chemistry, Electrochemistry

## Abstract

Inhibition of copper corrosion by some pyrimidinone derivatives, namely; (E)-*N*-(3-((1,3-dimethyl-2,4,6-trioxohexahydropyrimidin-5-yl)diazenyl)-2,5-diethoxyphenyl)benzamide (MA-975) and(E)-6-(4-((4-chlorophenyl)diazenyl)-3-methyl-5-oxo-4,5-dihydro-1*H*-pyrazol-1-yl)-1,3 dimethylpyrimidine-2,4(1*H*,3*H*)-dione (MA-978C) in 1.0 M nitric acid (HNO_3_) was studied using weight loss (WL), electrochemical impedance spectroscopy (EIS), and potentiodynamic polarization (PP) measurements. The efficiency of inhibition increases as the concentration of inhibitor increases, and it also increases as the temperature increases. With the addition of the examined inhibitors, significant corrosion protection was obtained, and (MA-975) showed a very promising % IE (89.59%) at 21 × 10^−6^ M using the (WL) method. The polarization data revealed that these compounds act as mixed-type compounds and are adsorbed on the copper surface following Langmuir adsorption isotherm forming a protective thin film protecting the metal in the corrosive media. Scanning electron microscopy (SEM) and Energy Dispersive X-ray were used to examine the surface morphology of copper samples. Quantum calculations and Monte Carlo simulation techniques were applied with informative yields and the results matched the experimental findings.

## Introduction

Corrosion is an essential process to consider in terms of safety and economics, especially for metals. Copper is a moderately noble metal^1–3^, and it has great electrical and thermal conductivities, as well as good corrosion resistance and workability. It is used in a lot of heating and cooling systems also it is utilized broadly as a part of industry, because of its warm conductivity. Copper’s major problem is its strong response to the acidic solutions. Several acid solutions (HCl, H_2_SO_4_, and HNO_3_) are it is utilized to remove harmful rust in various industrial operations. Among the commercially available acids, the most frequently used one is HNO_3_. The utilized corrosion protective of Cu in acid medium is mainly to less the corrosion of Cu at the time of acid descaling and cleaning. Copper corrosion depends on the way of nature as well as the state of utilization of materials. The most used method for copper corrosion protection is the utilization of organic inhibitors. Numerous organic inhibitors utilized are either created from low-cost raw materials or selected from compounds having hetero atoms in their long-chain carbon or aromatic system^4^. Most organic inhibitors are costly, toxic, and have a negative effect on the corrosion of metal. As a result, it is important and necessary to improve environmentally safe and low-cost corrosion protection^5^. The majority of the understood acid inhibitors are organic heterocyclic compounds containing O, S, P and/or N atoms^6–9^, these atoms enhance the activity of corrosion inhibitor. Adsorption of an inhibitor facilitates the protection of the metal surface^10^. As a result, covalent bonding (chemisorption) and/or electrostatic interaction (physisorption) can be used to create an adsorption coating on metal, protecting it against corrosive species attack^11^. It has been thought that it would be more desired and significant to produce novel low- or non-toxic corrosion inhibitors because the majority of conventional organic inhibitors are dangerous to the environment and people^12^. The presence of these functional groups and hetero-atoms in organic compound molecules promotes their action as copper corrosion inhibitors because these functional groups and hetero-atoms induce chemisorption. Organic derivatives also have a unique aptitude for inhibiting metal corrosion in acidic media^13^ and other solutions. The protection efficiency of organic inhibitors is primarily due to the presence of a polar group acting as an active center for adsorption on the metallic surface^14^. The presence of unoccupied d-orbitals in copper atoms that form coordinative bonds with atoms that can give electrons confirms this. There is also interaction with rings having conjugated bonds π-electrons. Heterocycle-containing pyrimidines have been reported to be safe inhibitors with excellent corrosion inhibition actions on copper metal in acidic media^15^. The corrosion inhibition properties of two pyrimidinone compounds were investigated in this study, coded MA-975 and MA-978C which clearly indicates that these derivatives work well as corrosion inhibitors, and that further research is recommended. Furthermore, considering recent environmental issues, these compounds are of significant concern due to their non-toxic appearances and high solubility in the test solution, which promotes protection efficiency. Some pyrimidine derivatives were utilized as corrosion inhibitors for steel and other metals in HCl and H_2_SO_4_ solutions with their percent *IE* predicted in Table 1. The most popular method of examining inhibitors today is still dazzle filtration. Researchers have coupled molecular dynamics simulation with thickness utility hypothesis calculation to urge deep insights into the barrier component of natural substances in an effort to understand this problem^16^.

**Table 1:** List of pyrimidine derivatives utilized as corrosion inhibitors for various metals and alloys.

Pyrimidine derivatives	Sample	Medium	%IE	References
a 2,6-dimethylpyrimidine-2-amine, *N*-Benzylidene-4,6-dimethylpyrimidine-2-amine and 2-[(3,6-dimethylpyridimine-2-ylimino) methyl]-4-nitrophenol in 2 M HCl; thymine, uracil, thymidine, and uridine	Mild steel	0.5 M HCl	96–97 at 10 mM	^17^
a) 6‐methyl‐4‐morpholin‐4‐yl‐2‐oxo‐1,2,3,4‐tetrahydro‐pyrimidine‐5‐carboxylic acid ethyl ester	Carbon steel	0.5 M HCl	80–86 at 0,25 g/L	^18^
b) 6‐methyl‐4‐morpholin‐4‐yl‐2‐thioxo‐1,2,3,4‐tetrahydro‐pyrimidine‐5‐carboxylic acid ethyl ester c) 6‐methyl‐4‐morpholin‐4‐yl‐2‐oxo‐1,2,3,4‐tetrahydro‐pyrimidine‐5‐carboxylic acid hydrazide d) 6‐methyl‐4‐morpholin‐4‐yl‐2‐thioxo‐1,2,3,4‐tetrahydro‐pyrimidine‐5‐ carboxylic acid hydrazide	Mild steel	0.5 M HCl	80–86 at 0.25 g/L	^18^
5-Benzoyl-4-(4-carboxphenyl)-6-phenyl-1,2,3,4-tetrahydro-2-iminopyrimidine, 5-benzoyl-4-tolyl-6-phenyl-1,2,3,4-tetrahydro-2-thioxopyrimidine in 1 M HCl	Stainless steel	1 M HCl	90 at5 × 10^-3^ M	^19^
5-benzoyl-4-(substituted phenyl)-6-phenyl-3,4-dihydropyrimidine-2(1*H*)- (thio)ones in 0.5 M H_2_SO_4_	Stainless steel	0.5 M H_2_SO_4_	92 at 2 × 10^–3^ M	^20^
a)5-(4-methoxyphenyl)-1,3,5,6,8-pentahydro-7-thioxo-pyrimido[4,5-*d*] pyrimidine-2,4-dione, b) 5-phenyl-1,3,5,6,8-pentahydro-7-thioxo-pyrimido[4,5-d] pyrimidine-2,4-dione, c) 5-(4-methoxyphenyl)-1,3,5,6,8-pentahydro-pyrimido[4,5-d] pyrimidine-2,4,7-trione d) 5-phenyl-1,3,5,6,8-pentahydro-pyrimido[4,5-*d*] pyrimidine-2,4,7-trione in HCl:	Mild steel	1 M HCl	97.1–88.0 at 400 ppm	^21^
1-(7-methyl-5-morpholin-4-yl-thiazolo[4,5-d] pyrimidin-2-yl)-hydrazine	Carbon steel	0.5 M H_2_SO_4_		^22^
a) 4,6-diphenyl-3,4-dihydropyrimidine-2(1*H*)-thione b) 4-(4-methylphenyl)-6-phenyl-3,4-dihydropyrimidine-2(1*H*)-thione c) 4-(4-methoxy-phenyl)-6-phenyl-3,4-dihydropyrimidine-2(1*H*)-thione	Carbon steel	1 M H_2_SO_4_	99–98 at 10 mM	^23^
a) 4-(4´-methylphenyl)-6-(phenyl)-3,4-dihydropyrimidine-2(1*H*)-thione b) 4-(4´-methoxylphenyl)-6-(phenyl)-3,4-dihydro-pyrimidine-2(1*H*)-thione in 2.0 M H_2_SO_4_^39^ for stainless steel 304	Stainless steel 304	2 M H2SO4	97.8, 96.2 at 5 mM	^24^
(3a, MA-1230), (3b, MA-1231) and (3c, MA-1232)	Copper	1 M HNO_3_	90.3–92.1 at 21 µM	^25^
(i) Ethyl(2-amino-5-methyl[1,4]-triazolo[1,5-a] pyrimidin-7-yl) acetate (ii) Ethyl (5-methyl[1,2,4] triazolo[1,5-a] pyrimidin-7-yl)-acetate	Mild steel	1* M* HCl	84, 85, respectively at 10^–3^ M	^26^
D-Glucose derivatives of dihydropyrido-[2,3-d:6,5-d0]-dipyrimidine-,4,6,8(1H,3H,5H,7H)- tetraone: GPH-3, GPH-2, GPH-1	Mild steel	1* M* HCl	93.9–97.8 at 10.15 × 10^-5^ M	^27^
3-(2-(4-(Hydroxymethyl)-1H-1,2,3-triazol-1-yl) ethyl)-2-methyl-6,7,8,9-tetra-hydropyrido [1,2-a] pyrimidin-4-one	Mild steel	1 M HCl	91 at 5 mM	^28^
Losartan potassium (LP) drug	Q235 steel	1 M HCl	92.0 at 5 mM at 318 K	^29^
5-(Benzylthio)-1H-tetrazole (BTTA), 5-Benzyl-1H-Tetrazole (BTA)	Q235 steel	0.5 M H_2_SO_4_	98.3% BTTA , 21.6% BTA for 2 mM	^30^

Our objective is to study more about the inhibitory action of these pyrimidinone compounds because these derivatives have high molecular size, low toxicity (safe) and contain O, N, CH_3_ groups and benzene rings. Some advanced techniques are used, SEM and EDX were employed to examine the surface, in addition to the application of quantum chemical calculations and Monte Carlo simulation was applied also.

## Materials and techniques

### Composition of copper samples

The chemical composition of the copper alloy used in this paper is as follows: (weight %) 0.0050 Zn, 0.0023 Pb, 0.0023 P, 0.0018 Co, 0.019 Fe, 0.0015 Si, 0.004 Ni, 0.0011 S.

### Solutions

The aggressive solution 0.5 M HNO_3_ was made by diluting analytical grade HNO_3_ (69%) with bi-distilled water, and all WL tests were performed in 100 ml unstirred solutions. Every studied compound's 100 ml stock solutions (10^–3^ M) were prepared by dissolving an accurately weighed quantity of each compound in an appropriate volume of dimethyl formamide (DMF) and absolute ethanol, then diluting with bi-distilled water to the needed concentrations (5 × 10^–6^–21 × 10^–6^ M).The solutions of these compounds are completely soluble in HNO_3_ due to it can be protonated in acid medium.

### Inhibitors

**Figure 1:**
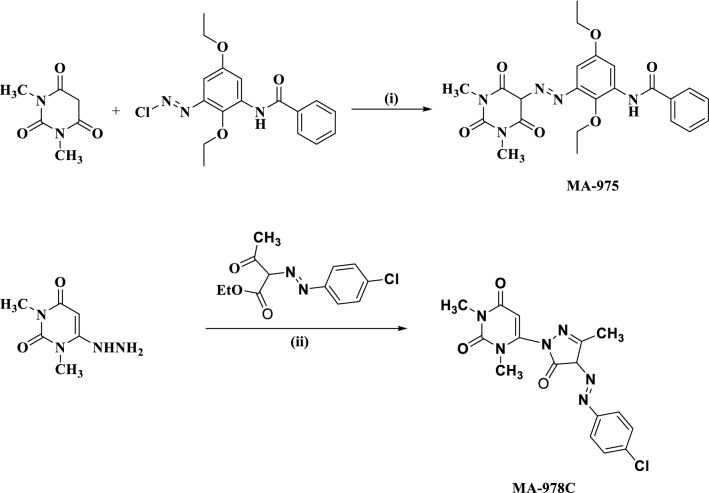
The identified pyrimidinone derivatives' synthetic pathway MA-975 and MA-978C, reagents and conditions: (i) AcONa, H_2_O, DMSO, 5–10 °C; (ii) EtOH/AcOH, reflux.

The synthesis of two pyrimidinone inhibitors was depicted in Fig. 1 as reported previously^31^ and their chemical structures and molecular formulas were listed in Table 2. The investigations of the two pyrimidinone compounds were done at different concentrations (5 × 10^−6^, 9 × 10^−6^, 13 × 10^−6^, 17 × 10^−6^ and 21 × 10^−6^ M) in the attendance and non-existence of the investigated compounds. In thermostatic conditions, all experiments were conducted.

**Table 2:** Pyrimidine-bichalcophene derivatives' molecular structures, formulae, and weights.

Inhibitor code	Molecular structures/ names	Mol. formulas (Mol. wt.)
MA-975	(*E*)-*N*-(3-((1,3-dimethyl-2,4,6-trioxohexahydropyrimidin-5-yl) diazenyl)-2,5-diethoxyphenyl) benzamide	**C**_**23**_**H**_**25**_**N**_**5**_**O**_**6**_ (467.18)
MA-978C	(*E*)-6-(4-((4-chlorophenyl) diazenyl)-3-methyl-5-oxo-4,5-dihydro-1*H*-pyrazol-1-yl)-1,3-dimethylpyrimidin-2,4(1*H*,3*H*)-dione	**C**_**16**_**H**_**15**_**ClN**_**6**_**O**_**3**_ (374.09)

### Corrosion methods

#### WL method

In corrosion investigations, WL measurements were utilized to select the best corrosion inhibitor^32^. The ASTM standard G 31–72^31^ was used to take this measurement. Mechanical polishing was done on the Cu alloy samples using various grades of emery sheets (1/0, 2/0, 3/0, 4/0, 5/0, and 6/0). The specimens are carefully cleaned with bi-distilled water and then degreased with acetone, dried at room temperature and then weighed. The test pieces were suspended by appropriate glass hooks at the beaker's edge and about 1 cm below the surface of the test fluid. The pieces were removed after a set amount of time, washed with bi-distilled water, dried, and weighed again. For three hours, different concentrations of inhibitors were tested for corrosion. 100 ml of blank without inhibitor and 1 M HNO_3_ with inhibitor were used to create these concentrations. The average value was calculated after the experiment was repeated three times. The percent *E*_*w*_ of the different solutions is calculated using the WL values acquired after a specified period in the mathematical Eq. (1):

1 $$\% E_{w} = \theta \times 100 = [1 - \left( {\Delta W/\Delta W^{\circ} } \right)] \times 100$$

where *ΔW* and *ΔW°* are weight losses per unit area (mg.cm^−2^) in the presence and the absence of the tested inhibitors, respectively.

#### Potentiodynamic polarization (PP) tests

A three electrode cell assembly with copper as the working electrode, a saturated calomel electrode (SCE) as the reference electrode, and platinum foil as the counter electrode was employed for these electrochemical research. The test sample was given 30 min to attain the steady state value of OCP before beginning the electrochemical studies. The potential range was (-200 to + 200 mV vs. SCE) at OCP with a scan rate of 0.1 *mVs*^*-1*^. The i_corr_ calculation was used to calculate the E_p_ and the θ from the following Eq. (2):

2 $$\% E_{p} = \theta \times 100 = \left[ {1 - \left( {i_{corr} /i_{corr}^{\circ} } \right)} \right] \times 100$$

where *i*_*corr*_ and *i*^*o*^_*corr*_ are the corrosion current densities with and without inhibitors, respectively.

#### Electrochemical impedance spectroscopy (EIS) tests

EIS measurements were carried out by using *AC* signal of 10 mV amplitude for the frequency spectrum from 100 kHz to 0.01 Hz. A Gamry Potentiostat / Galvanostat / ZRA" was utilized in electrochemical (PP & EIS) investigations (PCI4-G750). The *DC*105 *DC* Corrosion Program, the *EIS300* EIS Program, and a data collection computer are all part of Gamry. Echem Analyst version 5.5 was used to plot and calculate data.

### Surface morphology investigation by (SEM and EDX) analysis

The applied technique to study metal surface morphology are SEM and EDX it is used to give data about the surface morphology of Cu coins with and without highest degree of concentration of the three pyrimidine inhibitors utilizing (*SEM* model *JOEL, JSM-T20*, Japan). An energy dispersive X-ray (EDX) spectroscopy device was also used to evaluate the copper samples (Zeiss Evo 10 instrument model). The beam accelerating voltage was 25 kV.

### Quantum chemical calculations

All the quantum chemical tests occurred with finishing geometry similarity utilized Accelrys Material Studio.

### Quantum Monte Carlo simulation

MC is a molecular dynamics method based on classical mechanics and is one of the most broadly used theoretical techniques to describe the interaction between metal and inhibitor because it provides some essential parameters such as total energy, adsorption energy, and rigid adsorption energy. In our present study, the Monte Carlo simulation calculation was used to find the lowest energy for the tested system. The outputs and descriptors achieved by the Monte Carlo simulations, such as the whole adsorption, adsorption energy, firm adsorption, and deformation energies represent the most stable low energy configuration for the adsorption of the studied pyrimidine inhibitors on Cu (111) surface obtained through the Monte Carlo simulations ^33^.

## Results and discussion

### WL measurements

#### Effect of concentrations and temperature

**Figure 2:**
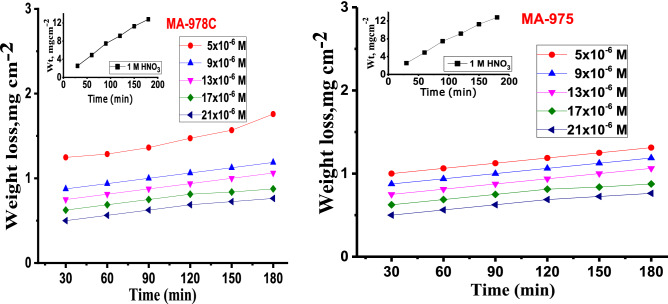
WL- time curves for dissolution of Cu alloy in 1 M HNO_3_ at different pyrimidine derivative concentrations (MA-978C and MA-975) at 25 °C.

The corrosion rate of copper alloy in nonexistence and existence of various concentrations of pyrimidine derivatives (5 × 10^–6^ M to 21 × 10^–6^ M) in 1.0 M HNO_3_ at 25–45 °C was studied. WL-time curves are graphically represented in Fig. 2 of pyrimidine derivatives. Corrosion parameters derived from WL method at different temperatures are given in Table 3, illustrate the calculated values of corrosion rate k_corr_ (mg.cm^-2^.min^-1^), *(%E*_*w*_*)* and the (θ) for copper alloy dissolution from which a reduction in k_corr_ is noticed when copper alloy the concentration of pyrimidine derivatives increases. This behavior can be explained based on the strong interaction among pyrimidine derivatives and copper alloy surface^34^**.** In general, when the concentration of these pyrimidine derivatives rises, the value of (percent *E*_*w*_) rises as well. These findings offer evidence that pyrimidines are effective inhibitors for Cu alloy against dissolution^35^. By increasing temperature, this lead to increase adsorption of inhibitor molecules on the alloy surface and some chemical changes in the inhibitor molecules may occur, resulting in an increase in electron densities at the adsorption centers of the molecules. There is a slight decrease in the corrosion rate *k*_*corr*_ of pyrimidine derivatives^13^ and hence the *%E*_*w*_ reaches (89.59%) at the optimum concentration (21 × 10^–6^ M). The trend of %E_w_ follows the order MA-975 > MA-978C.

**Table 3:** *k*_*corr*_ and *%E*_*w*_ of pyrimidine derivatives at various temperatures.

Temp., ^o^C	Conc, × 10^6^ M	MA-978C		MA-975	
		*k*_*corr*_ (mg cm^−2^ min^−1^)	*%η*	*k*_*corr*_ (mg cm^−2^ min^-1^)	%*η*
25	Blank	0.052 ± 0.0021	–	0.052 ± 0.0021	–
	5	0.0179 ± 0.0015	65.6	0.0157 ± 0.0012	69.8
	9	0.0172 ± 0.0023	66.9	0.0125 ± 0.0014	76.0
	14	0.0156 ± 0.0026	70.0	0.0122 ± 0.0021	76.5
	17	0.0138 ± 0.0009	73.5	0.0116 ± 0.0020	77.7
	21	0.0088 ± 0.0019	83.1	0.0087 ± 0.0019	83.3
30	Blank	0.089 ± 0.0022	–	0.089 ± 0.0022	–
	5	0.0305 ± 0.0009	65.7	0.0211 ± 0.0017	76.3
	9	0.0242 ± 0.0023	72.8	0.0192 ± 0.0023	78.4
	14	0.0208 ± 0.0017	76.6	0.0179 ± 0.0020	79.9
	17	0.0178 ± 0.0026	80.0	0.0153 ± 0.0018	82.8
	21	0.0145 ± 0.0021	83.7	0.0136 ± 0.0021	84.7
35	Blank	0.101 ± 0.0015	–	0.101 ± 0.0015	–
	5	0.0318 ± 0.0017	68.5	0.0216 ± 0.0002	78.6
	9	0.0209 ± 0.0020	79.3	0.0202 ± 0.0023	80.0
	14	0.0181 ± 0.0021	82.1	0.0169 ± 0.0017	83.3
	17	0.0173 ± 0.0012	82.9	0.0108 ± 0.0018	89.3
	21	0.0161 ± 0.0017	84.1	0.0123 ± 0.0015	87.8
40	Blank	0.137 ± 0.0020	–	0.137 ± 0.0020	–
	5	0.0420 ± 0.0020	69.3	0.0274 ± 0.0017	80.0
	9	0.0276 ± 0.0023	79.9	0.0252 ± 0.0020	81.6
	14	0.0223 ± 0.0018	83.7	0.0205 ± 0.0018	85.0
	17	0.0209 ± 0.0026	84.7	0.0182 ± 0.0023	86.7
	21	0.0154 ± 0.0023	88.8	0.0151 ± 0.0020	89.0
45	Blank	0.151 ± 0.0015	–	0.151 ± 0.0015	–
	5	0.0379 ± 0.0023	74.9	0.0302 ± 0.0010	80.0
	9	0.0259 ± 0.0021	82.9	0.0257 ± 0.0015	83.0
	14	0.0227 ± 0.0013	85.0	0.0214 ± 0.0017	85.8
	17	0.0197 ± 0.0017	87.0	0.0194 ± 0.0020	87.2
	21	0.0164 ± 0.0018	89.1	0.0157 ± 0.0012	89.6

#### Thermodynamic activation parameters

**Figure 3:**
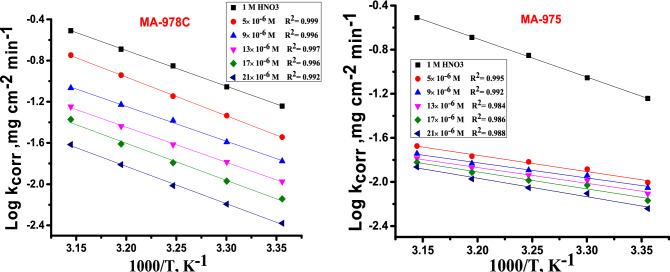
Arrhenius diagrams for Cu dissolution in the 1 M HNO_3_ solution of pyrimidine derivatives (MA-978C, MA-975).

**Figure 4:**
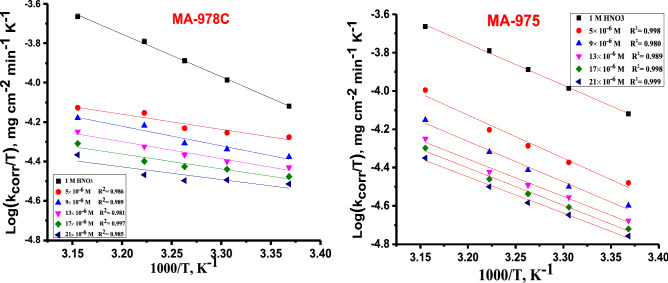
Transition state diagrams for Cu dissolution in the 1 M HNO_3_ solution of pyrimidine derivatives (MA-978C, MA-975).

Corrosion reactions obey Arrhenius processes and the apparent activation energy, E^*^_a_, for corrosion of copper alloy in 1 M HNO_3_ solution in the existence, and absence at different concentrations of pyrimidine derivatives at 25–45 °C was calculated from Arrhenius Eq.3:

3 $$\log k_{corr} = \left( {\frac{{ - E_{a}^{*} }}{{2.303{\text{R}}T}}} \right) + \log A$$

where ${{\varvec{E}}}_{{\varvec{a}}}^{\boldsymbol{*}}$ is the apparent activation energy of copper alloy dissolution and A is the pre-exponential factor, R is the molar gas constant, T is the absolute temperature. Plots of log *k*_*corr*_ vs. 1/T are displayed as straight lines for the tested inhibitors and the E^*^_a_ was calculated from the slope of these lines Fig. 3. It can be seen from Table 4 the data that ${{\varvec{E}}}_{{\varvec{a}}}^{\boldsymbol{*}}$ values for the inhibitors attains lower values for inhibitor-containing solutions. Similar results have been obtained in the corrosion inhibition of copper alloy and the decrease in ${{\varvec{E}}}_{{\varvec{a}}}^{\boldsymbol{*}}$ signifies chemisorption^36^. The transition state equation (4) was used to calculate other important thermodynamic activation parameters^36^:

4 $${\text{log k}}_{{{\text{corr}}}} = \log \left( {\frac{{\text{R}}}{{{\text{Nh}}}}} \right) + \frac{{\Delta {\text{S}}^{*} }}{{2.303{\text{R}}}} + \frac{{\Delta {\text{H}}^{*} }}{{2.303{\text{RT}}}}$$

where, *k*_*corr*_ denotes to the corrosion rate, R is the molar gas constant, ΔH^*^ and ΔS^*^ is the enthalpy and entropy of activation, respectively. Plots of log *k*_*corr*_*/T* vs 1000/T in Fig. 4 for the two derivatives, from which the values of ΔH* and ΔS*were calculated and are recorded in Table 4 for the investigated inhibitors Fig. 4 shows ${{\varvec{E}}}_{{\varvec{a}}}^{*}$ for blank solution is 68 0.71 kJ mol^−1^ and it decreased with increasing the concentrations of pyrimidine inhibitors. The decreased value of ${{\varvec{E}}}_{{\varvec{a}}}^{\boldsymbol{*}}$ when inhibitors are present is owing to their adsorption on the Cu surface. The chemisorption process^37^ has this tendency. When ΔH^*^ reaches a positive value, the corrosion process is endothermic, and it is classified as chemical adsorption. The activation entropy ∆S^*^ is large and negative, showing that the creation of an activated complex in the transition state involves association rather than dissociation, and that there is a decrease in disorder as reactants transform into the activated complex ^38^.

**Table 4:** Activation parameter for Cu dissolution in 1.0 M HNO_3_ in the absence and presence of (MA-978C and MA-975).

Inhibitor	Conc., × 10^–6^ M	Activation parameters		
		*E*_a_*	ΔH *	− ΔS*
		kJ mol^−1^	kJ mol^−1^	J mol^-1^ K^-1^
Free Acid (1 M HNO_3_)		68.71 ± 0.2028	88.29 ± 0.2403	175 ± 0.2504
MA-978C	5	29.06 ± 0.2309	43.05 ± 0.1453	139 ± 0.2309
	9	27.81 ± 0.1732	40.46 ± 0.2309	150 ± 0.2027
	13	26.96 ± 0.2333	37.37 ± 0.2729	161 ± 0.2603
	17	26.05 ± 0.2404	37.36 ± 0.1453	162 ± 0.1732
	21	25.19 ± 0.1453	36.24 ± 0.1453	167 ± 0.2333
MA-975	5	67.51 ± 0.2028	74.31 ± 0.1856	200 ± 0.2333
	9	65.60 ± 0.1528	79.62 ± 0.1764	205 ± 0.2333
	13	64.92 ± 0.2603	76.69 ± 0.1528	211 ± 0.1856
	17	63.57 ± 0.2048	76.31 ± 0.1453	223 ± 0.1764
	21	62.68 ± 0.2028	61.89 ± 0.2646	224 ± 0.1453

### Adsorption study

Isotherms provide significant information for understanding the nature of the corrosion inhibition process. The best fit was found using the Langmuir isotherm, which created a linear relationship between *θ *values and inhibitor concentration C. The Langmuir model asserts that the metal surface has a definite proportion of adsorption sites with one adsorbate, and the Gibbs free energy of adsorption for the sites is the same, regardless of the value of *ϴ.* This adsorption isotherm is described by the following equation Eq. (5) ^39^

5 $$\frac{C}{\theta } = \left( {\frac{1}{{{\mathbf{K}}_{{{\text{ads}}}} }}} \right) + C$$

where *K*_*ad*s_ is the equilibrium constant of adsorption process and C is the concentration of inhibitor and θ is the fraction of surface coverage. The *∆Gºads* of adsorption process was calculated from the following Eq. (6):

6 $${\mathbf{K}}_{{{\text{ads}}}} = \frac{1}{55}.5\exp \left( {\frac{{ - \Delta {\text{G}}_{{{\text{ads}}}}^{\circ} }}{{{\text{RT}}}}} \right)$$

Table 4 displays that the *∆Gº*_*ads*_ attained slightly more negative values than -40 kJ mol^-1^ and increases slightly by raising the temperature. This indicates that pyrimidine inhibitors may adsorbed physically or chemically on Cu surface, but the chemical adsorption is acceptable than the physical one ^40^. Also, other important adsorption parameters were calculated such as the enthalpy *(ΔH*^*o*^_*ads*_*)* and the entropy *(ΔS*^*o*^_*ads*_*)* of adsorption by applying Vanʼt Hoff (7) and thermodynamic general Eq. (8) ^34^:

7 $$\ln {\mathbf{K}}_{{{\text{ads}}}} = \frac{{ - \Delta {\text{H}}^{\circ} }}{{{\text{RT}}}} + {\text{constant}}$$

8 $$\Delta G_{ads}^{\circ} = \Delta H_{ads}^{\circ} - T\Delta S_{ads}^{\circ}$$

**Figure 5:**
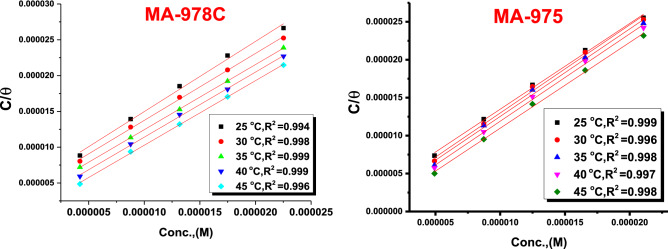
Langmuir diagrams for Cu dissolution in the 1 M HNO_3_ solution of pyrimidine derivatives (MA-978C, MA-975).

Table 5 reveals that positive values of *ΔH*°_*ads*_ indicate that pyrimidine derivative adsorption is an endothermic process^41^, implying that the inhibition efficiency increases as the temperature rises. This behavior can be explained by the fact that as the temperature rose, inhibitor molecules adhered to the metal surface^42^. In the presence of pyrimidine derivatives, the value of *ΔS*°_*ads*_ is positive because the endothermic adsorption process is always accompanied by a rise in entropy. The adsorption of inhibitor onto the Cu surface was propelled by an increase in entropy.^44^. Figure 5**s**hows Langmuir isotherm plots for the corrosion of Cu in the 1 M HNO_3_ with optimum concentrations of the investigated inhibitors.

**Table 5:** Parameters obtained from MA-978C, MA-975 adsorbed on the surface of the copper alloy in 1 M HNO_3_ acid at altered temperatures.

Inhibitor	Temp °C	− Δ*G°*_*ads*_ kJ mol^-1^	Δ*H*° _*ads*_ kJ mol^-1^	Δ*S*° _*ads*_ J mol^−1^ K^-1^
MA-978C	25	40.79 ± 0.2028	70.77 ± 0.1413	35.89 ± 0.2333
	30	42.25 ± 0.1453		37.92 ± 0.2028
	35	42.91 ± 0.1741		36.31 ± 0.1453
	40	44.14 ± 0.1732		37.92 ± 0.1732
	45	45.10 ± 0.2025		38.00 ± 0.2128
MA-975	25	40.11 ± 0.1000	50.4 ± 0.1673	30.37 ± 0.1453
	30	41.19 ± 0.1453		30.22 ± 0.1764
	35	42.57 ± 0.1732		301.8 ± 0.1453
	40	42.23 ± 0.1453		30.23 ± 0.1732
	45	43.03 ± 0.1732		30.32 ± 0.2028

#### Electrochemical impedance spectroscopy (EIS) measurements

**Figure 6:**
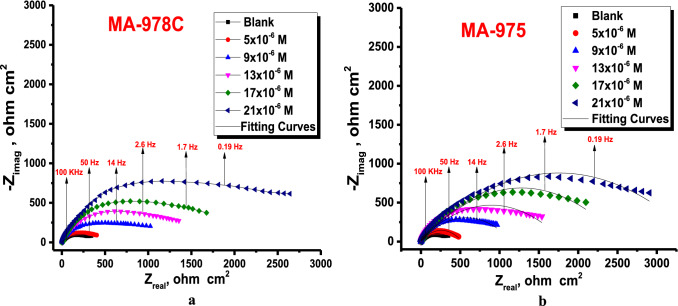
Nyquist bends for dissolution of copper metal in 1.0 M HNO_3_ attendance and nonattendance different concentrations of (**a**) MA-1978C, (**b**) MA-975 at 25 °C.

**Figure 7:**
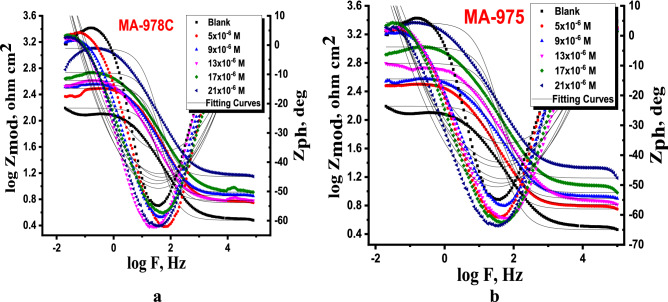
Bode bends for dissolution of copper metal in 1.0 M HNO_3_ attendance and nonattendance different concentrations of (**a**) MA-978C (**b**) MA-975 at 25 °C.

**Figure 8:**
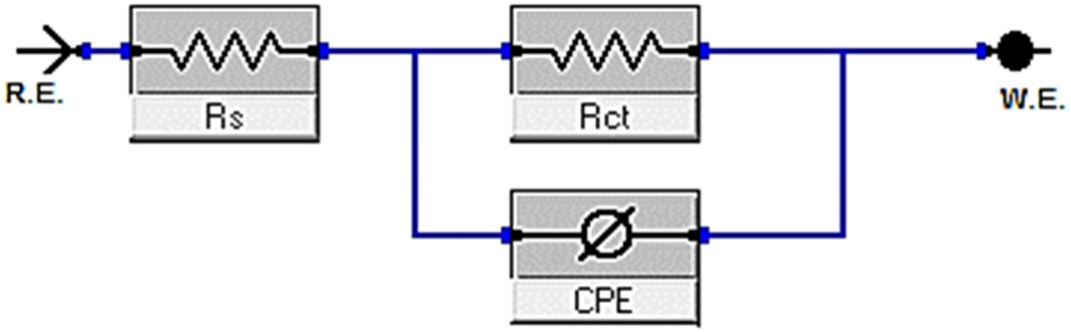
Equivalent circuit model for measuring EIS data.

The corrosion manners of copper alloy in 1 M HNO_3_ solution absence and presence of various doses of investigated pyrimidine was studied at 25 ± 1 °C. The impedance plots including the Nyquist (a) and Bode (b) are presented in Figs. 6 and 7. In the presence of inhibitor, the Nyquist diagram shows two-time constants: the capacitive loop at high frequencies and a straight line perceived as Warburg impedance at low frequencies. The charge transfer process can be related to higher frequency capacitive loops. Surface layer resistance, surface layer capacity, and the Warburg element, which signals a diffusion process across the surface layer, make up the second time constant in the low frequency range. With the addition of MA-978C and MA-975 to the Bode plots recorded in the presence of inhibitor, the Bode amplitude values over the entire frequency range increase. As further inspection in Fig. 7 in most cases of copper alloy corrosion in acid solution, the obtained Nyquist impedance diagrams in most cases does not show perfect semicircle, and this is arising from the frequency dispersion due to the roughness and heterogeneity of the electrode surface^42,43^. From EIS measurements, the impedance diagram is presented as a large capacitive loop with low frequencies dispersion^44^. The EIS spectra were analyzed by assuming CPE circuit and modeling the impedance data with the simplest equivalent electrical circuit (Fig. 8). In this equivalent circuit, the solution resistance *R*_*s*_ and the double layer capacitance *C*_*dl*_ are considered in parallel to the charge transfer resistance *Rct*^45^. The standard criteria for evaluation of pyrimidine derivatives best-fit were followed: The chi-square error was low (< 10^−3^) and the acceptable errors of elements in fitting (5%). In inhibited solution of 1.0 M HNO_3_ with various concentrations of inhibitors, the impedance diagrams follow the same pattern (one capacitive loop), however, the diameter of this capacitive loop increases with increasing concentration^46^. *“n”* is the CPE parameter which characterizes the deviation of the system from ideal capacitive behavior. The values *n* is between -1 and 1. For a perfect resistor, *n* = 0 and for an inductor *n* =-1 for solutions contain pyrimidine are higher than those obtained for pyrimidine free solution which reflects their inhibitive action for copper alloy dissolution process. The *(n)* value is a measure of a surface's roughness, and its rise in this study could indicate a drop in the heterogeneity of the working electrode surface due to inhibitor molecule adsorption and increases with inhibitor concentration, while the reverse is the case with *Y*_*o*_. The EIS parameters such as *R*_*ct*_*, R*_*s*_*, C*_*dl*_*,* and θ and % E_EIS_ were listed in Table 6, from which we can conclude that^47^, *R*_*ct*_ increases and ***Y***^***ο***^ decreases when the concentration of pyrimidine inhibitors increased and hence, the %E_EIS_ increases. This is due to the increased thickness of the double layer or a reduced dielectric constant. The decreased ***Y***^***ο***^ values recommended the decreased thickness of the oxide layer in the presence of inhibitors. The lowering in *C*_*dl*_*,* might be caused by a decrease in local dielectric constant and/or an increase in the thickness of the electrical double layer, indicates that the inhibitor molecules have adsorb at the metal/solution interface^48^. The increase of *R*_*s*_ with inhibitor concentration proofs the increase of the thickness of double layer. Also, single charge transfer process occurred during dissolution of copper alloy in 1.0 M HNO_3_ which does not change in the presence of investigated pyrimidine. This is indicated from the presence of single semicircle loop ^49^. The impedance of the CPE represented by the next Eq. (9):

9 $$Z_{CPE} = 1/Y^{0} \left( {j\omega } \right)^{n}$$

**Table 6:** Impedance parameters for copper alloy in 1 M HNO_3_ in presence and absence different concentrations of pyrimidine derivatives at 25 °C.

Inh	Conc., µM	*R*s, (Ω cm^2^)	*Y*^*ο*^ (μΩ^−1^ s^n^cm^−2^)	*n*	*C*_dl,_ ( µF cm^-2^)	*R*_ct,_ (Ω cm^2^)	${\%\eta }_{EIS}$	*χ*^*2*^
blank	–	1.839	576.2	0.963	316.2 ± 0.2333	68.2 ± 0.1453	–	0.000087
MA-978C	5	1.914	451.7	0.972	205.7 ± 0.1453	113.7 ± 0.1764	40.1	0.000341
	9	2.175	397.3	0.975	182.3 ± 0.1732	149.4 ± 0.2028	54.4	0.000653
	13	2.372	351.4	0.977	139.1 ± 0.1453	208.2 ± 0.2309	67.3	0.000453
	17	2.486	312.5	0.978	107.4 ± 0.2333	327.9 ± 0.1732	79.2	0.000654
	21	2.591	268.1	0.980	72.6 ± 0.1453	614.2 ± 0.1732	88.9	0.000745
MA-975	5	1.758	439.1	0.914	197.2 ± 0.1202	120.6 ± 0.2028	43.5	0.000065
	9	1.831	392.4	0.969	174.6 ± 0.2309	156.1 ± 0.1732	56.3	0.000451
	13	1.905	345.2	0.982	132.5 ± 0.2028	213.9 ± 0.1453	68.1	0.000123
	17	1.938	306.3	0.985	98.1 ± 0.1732	374.2 ± 0.2028	81.8	0.000231
	21	2.106	262.5	0.987	67.3 ± 0.1553	685.8 ± 0.2309	90.1	0.000129

Y^o^ is referred to CPE constant, j is referring to the imaginary root, ɷ is refer to the angular frequency, n (-1 < n < 1) stands for the deviation index.

The C_dl_ value was obtained from this eq. (10)^50^.

10 $$C_{dl} = \frac{1}{{2\pi f_{\max } R_{ct} }}$$

The corrosion %η was calculated by using the Eq. (11):

11 $$\% \eta_{EIS} = \left( {\frac{{R_{ct} - R_{ct}^{*} }}{{R_{ct} }}} \right) \times 100$$

where R^o^_ct_, and R_ct_ are the resistance of the charge transfer in the absence and presence of the tested compounds respectively.

#### Open circuit potential (E_OC_)

**Figure 9:**
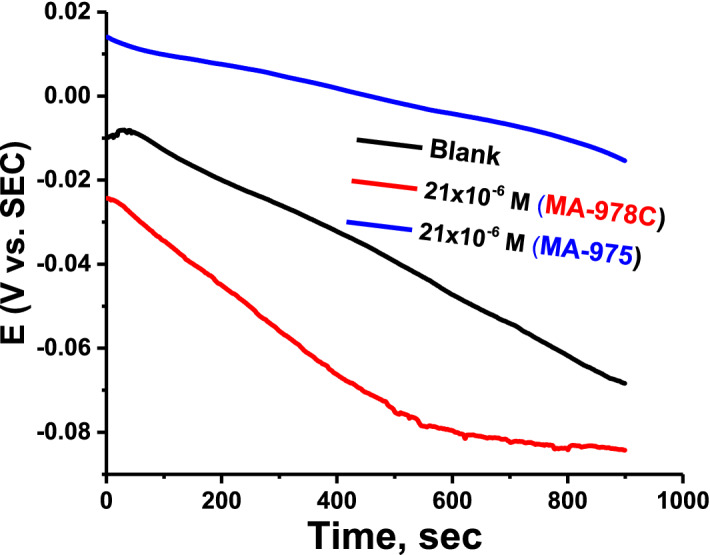
E_OC_ versus time at 25 °C for Cu in the acid corrosive medium in the absence and attendance dose of organic compound (21 × 10^–6^ M).

Figure 9 shows the E_OC_ fluctuation with time for copper in an acid corrosive media with no dose of organic component (21 × 10^–6^) present. Figure 9 shows that after 100 s, the blank solution stabilizes at a value of -7 mV/SCE. Cu oxidizes, resulting in the formation of corrosive products on its surface. As a result, when an organic compound is present at a dose of 21 × 10^–6^ M, the potential changes quickly and remains stable over time. The resistance of Cu dissolving in the acid corrosive media is indicated by the initial shifts in E_OC_ while adding concentrations of organic substance. The disintegration of the oxide coating and the formation of a protective film on the Cu surface can explain this phenomenon.

#### Potentiodynamic polarization (PP) measurements

**Figure 10:**
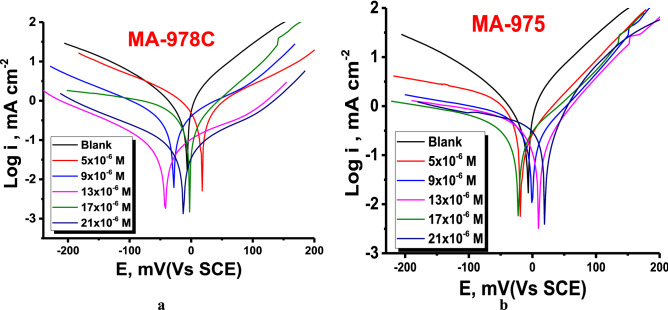
PP bends for copper alloy in the 1 M HNO_3_ solution at altered dose of pyrimidine derivatives (**a**) MA-978C, (**b**) MA-975 at 25 °C.

Tafel polarization curves of copper alloy in uninhibited and inhibited 1 M HNO_3_ solution with different concentrations of pyrimidine derivatives at 25°Care illustrated in Fig. 10. Cu exposed to an uncontrolled system corroded severely; this is due to the aggressive NO^-^_3_ ions, which drive strong Cu dissolution. Fortunately, the addition of pyrimidine analogues to a solution minimized Cu oxidation due to considerable reductions in *i*_*corr*_*.* The variation of PP parameters with the concentration of pyrimidine compounds are given in Table 7. The PP indicates that, the behavior of pyrimidine derivatives is of Tafel-type because the addition of pyrimidine compounds increases the cathodic and anodic potential with a displacement to more negative and positive values, respectively. The corrosion current density *(i*_*corr*_*)* drops as the concentration of pyrimidine increases, indicating that the presence of these compounds slows Cu dissolution and that the degree of inhibition is proportional to the concentration. The order of additives is $\mathrm{MA}-975>\mathrm{MA}-978\mathrm{C}$. The maximum difference in *E*_*corr*_ values between the inhibited and uninhibited systems was less than 39 mV, indicating that the investigated pyrimidine derivatives are mixed type inhibitors that affect both the cathodic and anodic polarization curves^51^. Moreover, the cathodic and anodic polarization Tafel lines keep a similar pattern and slightly changed by adding the pyrimidine derivatives. In other words, the nature of the polarization curves remains approximately the same irrespective of different concentration of these derivatives addition to acid solution which suggests that the mechanism of Cu in nitric acid does not change by adding these derivatives to the acid solution. The inhibitive action of pyrimidine compounds is initiated by adsorption on the reactive sites on the electrode surface and blocking corrosion cells and reducing the exposed surface area available for attack from corrosion environment^52^. The E_p_% was calculated next Eq. (12):

12 $$Ep\% = \left( {\frac{{i_{corr} - i^{\prime}_{{{\text{corr}}}} }}{{i_{corr} }}} \right) \times 100$$

where *i‵*_*corr*_ and *i*_*corr*_ refer to the corrosion current of the copper metal presence and absence pyrimidine derivatives, respectively. The results showed that i_corr_ reduces with increasing concentration up to 21 × 10^–6^ M and then significantly decreases when the concentration of pyrimidine derivatives reaches 25 × 10^–6^ M. As a result, when the concentration of pyrimidine compounds was raised to 21 × 10^–6^ M, the inhibitory performance improved. The addition of more pyrimidine derivatives than 21 × 10^–6^ M may lead to a slower kinetic at the adsorption sites, allowing the desorption of the adsorbed layer and hence lower inhibition efficiency.

**Table 7:** PP measurements for copper alloy in 1 M HNO_3_ with and without altered concentrations of the tested pyrimidine derivatives at 25 °C.

Conc, M × 10^6^	− *E*_corr._ mV (vs.SCE)	*i*_corr_, µA cm^−2^	*β*_a_, mV dec^−1^	− *β*_c_, mV dec^−1^	*θ*	*E*_*p*_%	
Blank		−7± 0.1453	352.7 ± .1732	86.4 ± 0.2309	145.2 ± 0.2028	**–**	**–**
MA-978C	5	19.1 ± 0.2028	209.3 ± 0.2028	77.7 ± 0.2028	191.4 ± 0.1453	0.407	40.7
	9	− 29.2 ± 0.2431	168.1 ± 0.1155	85.4 ± 0.1732	157.50.2028	0.523	52.3
	13	− 43.4 ± 0.2055	135.2 ± 0.2603	72.8 ± 0.2309	179.1 ± 0.2906	0.617	61.7
	17	− 2.5 ± 0.1452	87.4 ± 0.1764	91.3 ± 0.2333	190.1 ± 0.1732	0.752	75.2
	21	− 13.2 ± 0.1742	42.1 ± 0.2028	80.2 ± 0.1202	204.6 ± 0.2028	0.881	88.1
MA-975	5	− 18.8 ± 0.2102	201.4 ± 0.1732	89.9 ± 0.1732	144.2 ± 0.17638	0.429	42.9
	9	− 1.1 ± 0.2209	158.1 ± 0.1453	137.6 ± 0.1453	148.6 ± 0.2082	0.552	55.2
	13	10.2 ± 0.2010	121.7 ± 0.1732	186.6 ± 0.2027	155.4 ± 0.1732	0.655	65.5
	17	− 22.5 ± 0.1753	84.3 ± 0.2028	72.1 ± 0.2333	94.2 ± 0.2082	0.761	76.1
	21	19.1 ± 0.1208	35.7 ± 0.2010	124.1 ± 0.1764	139.9 ± 0.1764	0.899	89.9

### Surface analysis

#### Scanning electron microscope (SEM) and energy dispersive X-ray (EDX) analysis.

**Figure 11:**
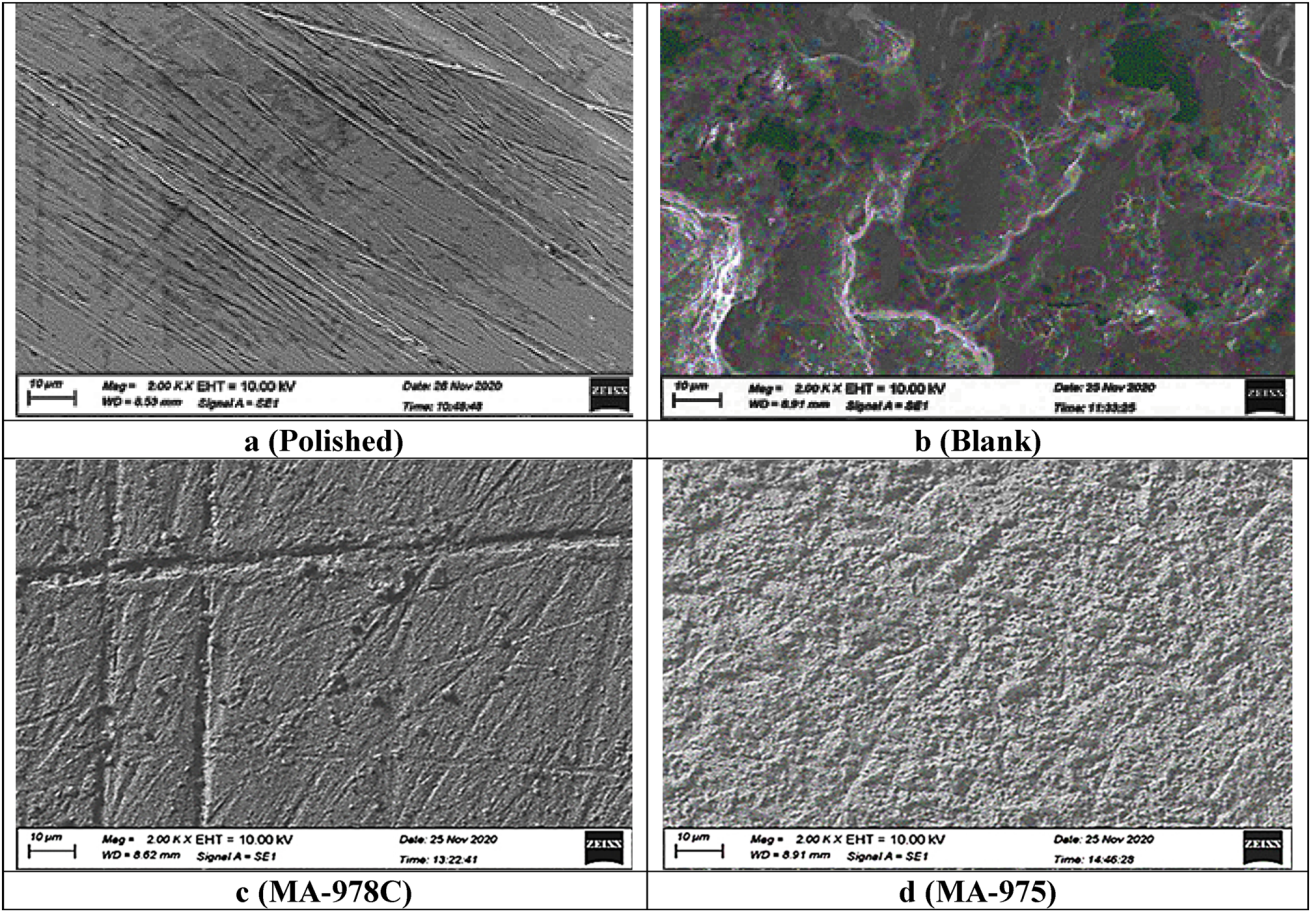
SEM images of copper alloy without (blank) and with 21 µM of MA-978C, and MA-975 at 25 °C.

**Figure 12:**
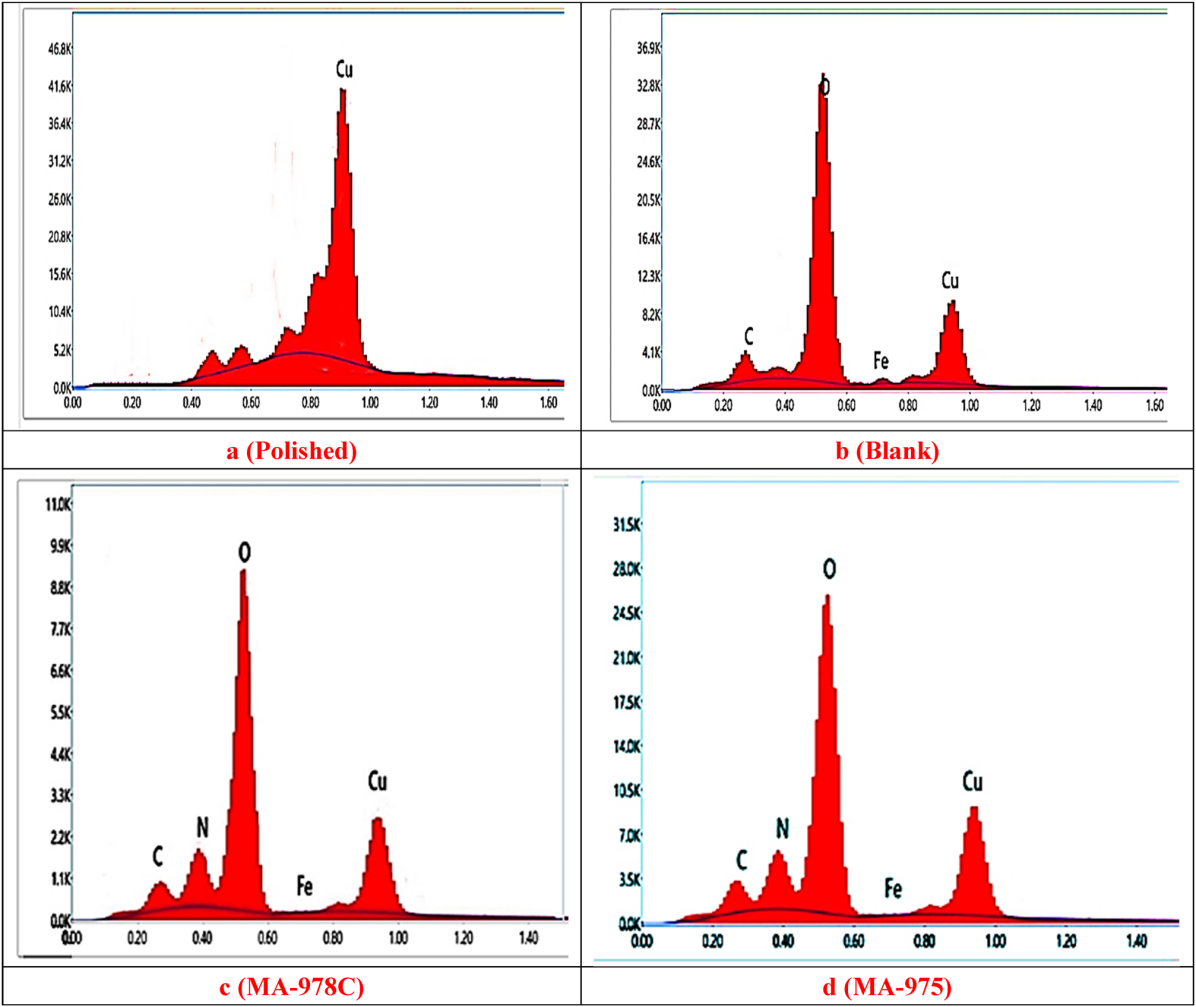
EDX images of Cu alloy without (blank) and with 21 µM of MA-978C, and MA-975 at 25 °C.

The morphology of the copper metal surface was investigated using a scanning electron microscope, when copper samples were immersed in 1 M HNO_3_ solution for 24 h with and without the tested pyrimidine derivatives^53^. The effect of these derivatives on the corroding copper metal surface is also analyzed by EDX with SEM images to investigate the pyrimidine derivatives adsorption by comparing the elements identified and detected on the copper metal surface with the elements in the pyrimidine derivatives molecular structure. Figures 11 and 12 show SEM and EDX images, respectively. According to EDX analysis, the copper surface is heavily damaged without the inhibited solution, where the images of inhibited copper surface indicated less corrosion in the presence of examined pyrimidine derivatives. Also, the percentage of iron in the copper surface immersed in inhibited solution is decreasing, while the percentage of the carbon and heteroatoms (S, O and N) is increasing^54^. From the SEM–EDX tests, we can conclude that the examined derivatives adsorbed on the copper metal surface show an excellent image for preventing severe corrosion of the metal surface^55^.

#### Quantum calculations

**Figure 13:**
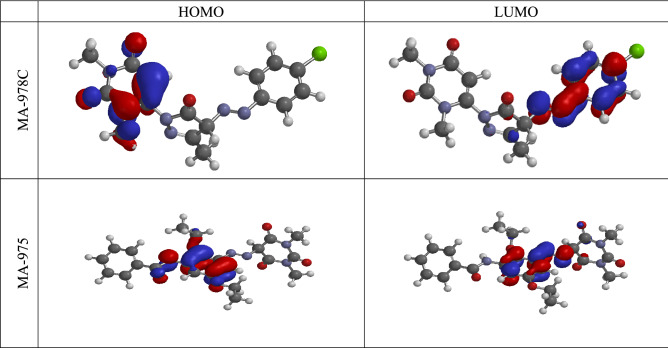
Molecular structure of the pyrimidine compound, and its frontier molecular orbital density distribution (HOMO and LUMO).

To determine whether there is a correlation between the molecular structures of investigated inhibitors and their inhibitory actions, quantum chemical simulations were conducted^46^. The synthesized MA-978C and MA-975 inhibitors' computed quantum chemical characteristics *(E*_*HOMO*_*, E*_*LUMO*_*,* and *ΔE*) were determined and are displayed in Table 8.The *E*_*HOMO*_*, E*_*LUMO*_ represented a molecule's capacity for electron donation and acceptance. These values may be seen in Fig. 13 as well. The ability of the inhibitor to give electrons was stronger the higher the E_HOMO_ value, whereas the ability of the inhibitor to receive electrons was stronger the lower the E_LUMO_ value^56^. A molecule's ability to deliver electrons to a suitable acceptor with unoccupied molecular orbitals is indicated by high values of E_HOMO_. The E_LUMO_, on the other hand, demonstrates a molecule's capacity to accept electrons; lower values correspond to a higher electron-accepting capacity.

**Table 8:** The calculated quantum chemical parameters for the investigated pyrimidine compounds.

Compound	MA-978C	MA-975
*E*_*HOMO*_, eV	− 9.43	− 8.92
*E*_*LUMO*_, eV	− 1.24	− 1.01
*ΔE*, eV	8.190	7.910
*I*_*P*_, eV	9.43	8.92
*E*_*A*_, eV	1.24	1.01
*η* , eV	4.095	3.955
*σ* , eV	0.244	0.253
*μ* , eV	5.335	4.965
Dipole moment (Debye)	2.930	3.220

The energy to eliminate an electron from the last orbital occupied is called the gap in the energy band *(ΔE* = *E*_*LUMO*_ − *E*_*HOMO*_)^57^. Low *ΔE* facilitates adsorption of the molecule and thus will cause higher inhibition efficiency, as *ΔE* decreases, the reactivity of the molecule increases leading to increase the inhibition efficiency of the molecule. The dipolar moment *(μ)* is a measurement of the polarity of the covalent bond between the compounds under examination Eq. (13). The high *μ* values are thought to improve the adsorption tendency of the compounds examined on metal surfaces. According to theoretical calculations, the band gap energy values ΔE for MA-975 is less than MA-978C, so the inhibition efficiency is supposed to be in the order MA-975 > MA-978C. This reflects a greater correlation between corrosion IE and this result^58^. The inhibitor (MA-975) has the lowest total energy, which suggests that its adsorption is higher with the highest softness, as shown in all the data in Fig. 13 and Table 8. In addition, the dipole moment is the parameter most used to describe the polarity of a molecule. It is clearly proved in the literature that molecules with high dipole moments are more reactive and hence, MA-975 > MA-978C^59^. Chemical properties that are important for determining molecular stability and reactivity include chemical hardness ($\eta$) (Eq. 15), which evaluates an atom's resistance to charge transfer, and softness ($\sigma$) (Eq. 16), which defines an atom or group of atoms' ability to accept electrons. From the results, as the chemical reactivity increases, the percent IE of adsorption increases, and the molecule with the lowest hardness value should have the highest inhibitory efficiency^60^. Because of the increase in softness *(σ* = 0.253, 0.224 eV-1 for MA-975, MA-978C, respectively) and reduction in hardness *((η)* = 4.095, 3.955 eV, for MA-975C, MA-978, respectively), the CBIPM inhibitor has high chemical reactivity with the metallic surface. The following equations were used to calculate the global hardness *(η),* softness *(σ),* and chemical potential *(µ)* in terms of IP and EA ^61^:

13 $$\mu = - \chi = - \frac{{I_{p } + E_{A} }}{2}$$

14 $$\chi = \frac{{I_{p } + E_{A} }}{2}$$

15 $$\eta = \frac{{I_{P - } E_{A} }}{2}$$

16 $$\sigma = \frac{1}{\eta }$$

The results show that the hetero atoms (N, S, and O) in the structure of compounds have a significant impact on quantum chemical parameters. This theoretically illustrates that the hetero atom (S) influences the adsorption of inhibitor chemicals on metal. We performed a molecular dynamics simulation on the inhibitors' adsorption on the copper surface.

#### Monte Carlo (MC) simulation

**Figure 14:**
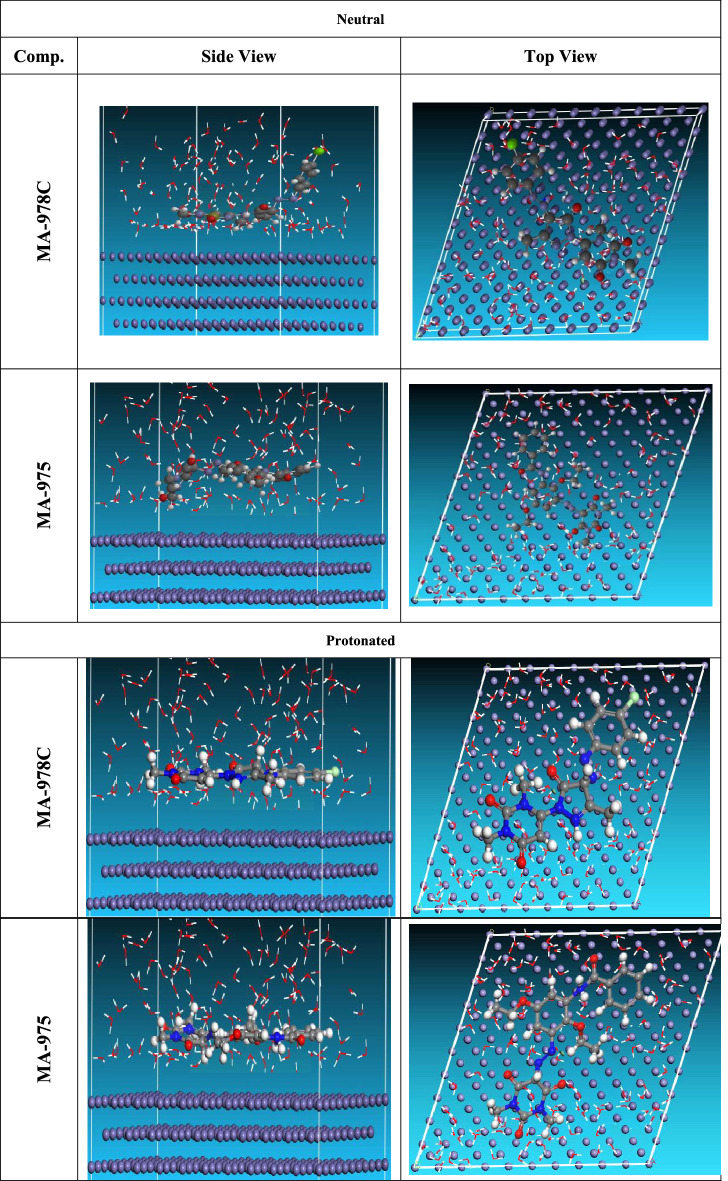
Equilibrium adsorption configurations of the inhibitors molecules on the Cu (111) surface: inhibitors side view and top view.

MC is an excellent technique for determining the most stable adsorption conformations of substituted pyrimidine derivatives in 1 M HNO_3_. Figure 14 shows the equilibrium adsorption configurations of the inhibitors molecules on the Cu (111) surface: inhibitors side view and top view. Different parameters derived from the Monte Carlo simulation shown in Table 9. The parameters contain total energy of the substrate–adsorbate outline. The sum of the rigid energy and the deformation energy is called to be the adsorption energy. In this study, the substrate energy is considered as zero. In addition, the energy of the adsorption in reports energy liberated when the relaxed adsorbate component is adsorbed on the substrate. The energy of the rigid adsorption reports the energy, liberated when the unrelaxed adsorbate components are adsorbed on the substrate, meaning that before the geometry optimization step. The deformation energy reports the energy, liberated when the adsorbed adsorbate constituents are relaxed on the substrate surface^62^. Table 9 shows also (dE_ads_/dNi), which reports the energy, of substrate–adsorbate configurations where one of the adsorbate components has been eliminated. As shown in Table 9, MA-975 and MA-978C Inhibitors gave high adsorption energy in negative value found during the simulation process. Furthermore, the protonated form of C_23_H_25_N_5_O_6_H^+^ and C_16_H_15_ClN_6_O_3_H^+^ molecules in corrosive solution have a higher negative adsorption energy than the neutral form, indicating that the protonated form of C_23_H_25_N_5_O_6_H^+^ and C_16_H_15_ClN_6_O_3_H^+^ molecules in corrosive solution has a positive impact on the corrosion protection process. Superior inhibition efficiency is typically correlated with high binding energy (E_binding_) between the inhibitor and alloy^63^. Inhibitor molecule equilibrium adsorption configurations on the Cu (111) surface for neutral and protonated side views and top views are shown in Fig. 14. (MA-975 > MA-978C) is the order of the manufactured inhibitors based on IE percent. It is obvious that both inhibitor molecules, in their various forms, adhere to the surface of Cu (111) in a nearly parallel pattern, covering the Cu alloy's maximum surface area. This behavior is primarily attributed to two inhibitor compounds' strong propensity to donate electrons to the vacant Cu orbitals and to accept electrons from copper's d-orbitals via a back-bonding^64^.

**Table 9:** Equilibrium adsorption configurations of the inhibitors molecules on the Cu (111) surface.

Structures		Adsorption energy	Rigid adsorption energy	Deformation energy	Compound dE_ad_/dNi	H_3_O^+^ dE_ad_/dNi	H_2_O dE_ad_/dNi
Neutral	Cu (111)/C_23_H_25_N_5_O_6_)	− 4095.203	− 4027.378	− 67.825	− 272.939	− 259.03	− 11.271
	Cu (111)/C_16_H_15_ClN_6_O_3_)	− 4090.983	− 4022.109	− 68.874	− 283.114	− 261.79	− 7.387
Protonated	Cu (111)/C_23_H_25_N_5_O_6_H^+^	− 4163.765	− 4083.24	− 80.525	− 281.869	− 262.64	− 12.361
	Cu (111)/C_16_H_15_ClN_6_O_3_H^+^)	− 4156.544	− 4074.97	− 81.574	− 293.044	− 265.28	− 9.478

#### Mechanism of corrosion inhibition

**Figure 15:**
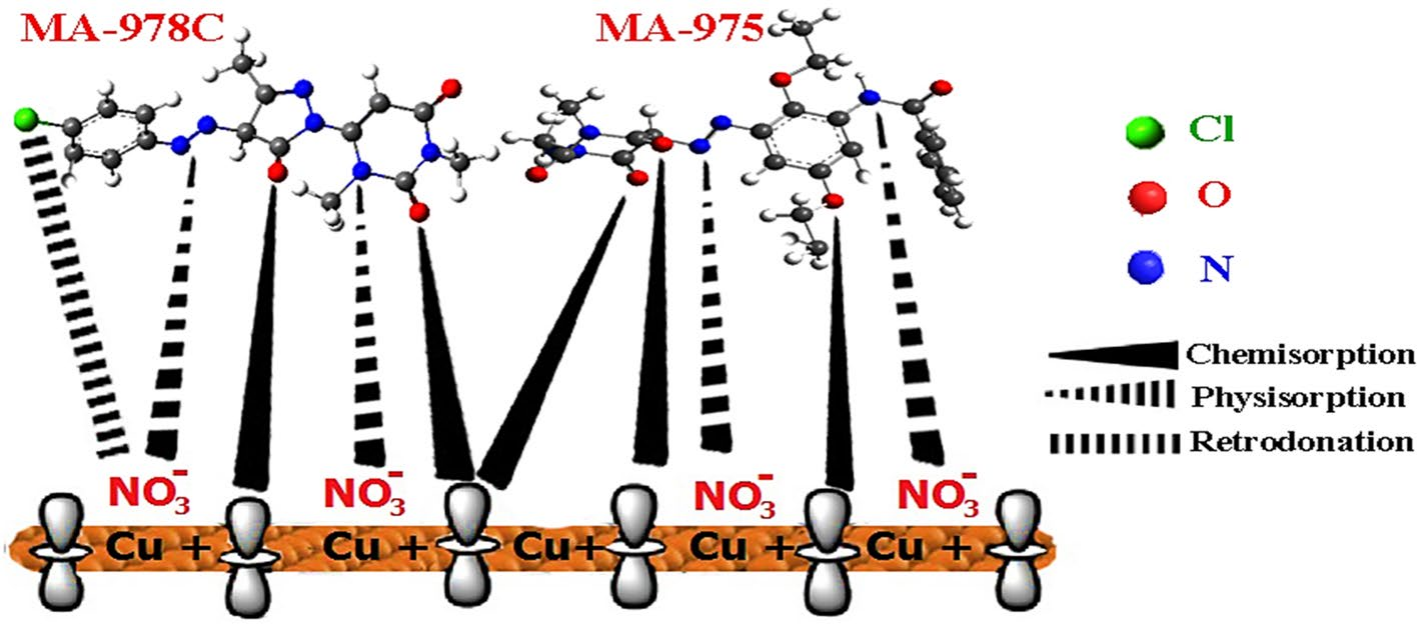
Mechanism of corrosion protection of Cu dipped in 1 M HNO_3_ media using MA-975, and MA-978C.

Physicochemical characteristics and the Cu charge may be utilized to illustrate adsorption based on the experimental study and theoretical calculations. Figure 15 depicts the inhibitory action of these pyrimidine derivatives on the surface of copper. Several studies have been revealed that the Cu surface in HNO_3_ solution is positively charged^65^. The presence of function groups capable of securely attaching the inhibitor molecules on the metal surface explains the inhibitor's propensity for being adsorbed on the metal surface. When the inhibitor concentration is raised, the formation of a protective coating of inhibitor molecules at the metal/solution interface improves inhibition effectiveness. The charge that is positive Cu surfaces encourage NO^3-^ adsorption, resulting in a negative charge surface that makes it simpler to adsorb cations in solution. These organic pyrimidine derivatives can be protonated in solution due to the unshared electron pairs of the N, O, and S electrons. Because of electrostatic interaction the protonated molecules were physisorbed on the metal surface. Meanwhile, as shown in Fig. 15, further adsorption of these inhibitors can be accomplished by forming covalent interactions (chemisorption). Based on quantum chemical measurements of both WL and electrochemical values, the percent IE of the two investigated pyrimidine derivatives is as follows: MA-975 > MA-978C. This is due to the following factors: MA-975 has a larger molecular size than MA-978C, allowing it to cover a larger area from the surface; MA-975 has 5 N and 6O atoms, but MA-978C contains 6 N, 3O, and one Cl, which is a withdrawing group, reducing the electron density on the molecule.

## Conclusion

1. The study revealed that the investigated pyrimidine derivatives were utilized as highly, safe efficient inhibitors for copper in 1 M HNO_3_.
2. The adsorption of the investigated inhibitors on copper surface follows the Langmuir adsorption isotherm. The development of a protective layer on the copper surface was confirmed using SEM and EDX analyses.
3. Tafel curves indicated that pyrimidine derivatives acted as a mixed-type corrosion inhibitor and provided superior inhibition performance for Cu corrosion in HNO_3_ medium at different temperatures, which was further confirmed by EIS method. The obtained IE values from EIS increased with increasing pyrimidine derivatives concentration which agreed well with those obtained from Tafel method.
4. The charge transfer resistance increases while the capacitance double layer drops by raising the inhibitor dosage which may be attributed to the adsorption of inhibitor molecules on the copper surface.
5. The high and low E_binding_ values indicated substantial pyrimidine derivative adsorption on copper surface. The pyrimidine derivatives' excellent capacity to donate and absorb electrons to/from copper was validated by the flat adsorption orientation of the compounds, creating an anchoring barrier to stop copper from corroding.
6. The obtained results of chemical, electrochemical and theoretical studies are in good agreement.

## Data Availability

Authors can confirm that all relevant data are included in the article.
